# In Memoriam—Dr. A. Judson Brewster

**Published:** 1897-04

**Authors:** 


### DR. A. JUDSON BREWSTER.

Dr. A. Judson Brewster, one of the oldest physicians of Syracuse, died at his home in Shonnard Street, Saturday, February 20th.

Dr. Brewster was born October 21st, 1824, at Ellisburg, Jefferson County, New York, and gained his early education at the district school and at Belleville Academy. He received his medical training at Hahnemann College, Philadelphia. In 1847 he was married to Ann E. Bartlett and moved soon afterward to Cato. Here he practiced medicine twenty-four years, and was a well-known and much respected citizen. In 1874 he removed to Syracuse, where he commanded the respect of the members of his profession and won the esteem of a wide circle of friends.

Dr. Brewster was a consistent and devout member of the Church of Christ from his early youth until death.—*Syracuse Post*, February 22d, 1897.

